# Near Approach to, and Devitalization of the Dental Pulp

**Published:** 1888-01

**Authors:** J. E Robinson

**Affiliations:** Cleveland


### Near Approach to, and Devitalization of the Dental Pulp.

BY J. E ROBINSON, D. D. S., CLEVELAND.

[Read before the Ohio State Dental Society, Springfield, Ohio, October, 1887.]

I am tempted to present a few thoughts inspired by reading some jottings of Dr. D. R. Jennings which were found lying unguarded on his desk one morning last summer. So nearly did his thoughts harmonize with my own experience that with his permission I have adopted many of them and inserted them here.

There has been a great deal of discussion during the past few years in regard to the best manner of treating exposed pulps, many still claiming that at least a very large per cent. can be saved alive, restored to perfect health, and made to perform all the functions of a pulp that has never been disturbed by exposure caused by decay, or by careless or accidental uncovering during the process of excavating.

So thoroughly and with such persistency was the certainty of being able to save the nerve alive by treatment, taught by most of our leading operators, that to deny it was simply to proclaim one's ignorance and acknowledge an inability to keep pace with the march of improvements in the profession. With many others the writer was led to make a few trials and with such seeming success that the nerve saving theory was fully adopted in his practice. But the Grim Reaper having spared him long enough to see the final results of the nerve saving, not only in his own treatments, but also in that of others who taught the doctrine, faith in the soundness of the nerve saving theory has been shaken. My observation leads to the conclusion that not one pulp in one hundred lives three years after exposure though the nerve be never so carefully and thoroughly treated and capped, and the cavity afterward excellently filled.

The value of the pulp and nerve is governed very largely by the age of the patient. Few persons over fifty years of age have any pulp; the nerve may be there but the pulp is gone, its mission having been completed and the space almost filled with calcific matter. In fact, of so little importance is either pulp or nerve to the usefulness of the tooth of a person advanced beyond middle life, that in many cases we find the entire canal obliterated or filled with a calcific deposit. Still it retains its sensitiveness in a degree to the action of the drill or excavator. I am therefore led to believe that in all cases where the patient has arrived at an age when the teeth show considerable wear or the gums have begun to recede, it is better to destroy as much of the exposed pulp and nerve as remain and fill the space; it is much more satisfactory to yourself and patient The uses of the pulp and nerve filaments are to assist the development by furnishing nourishment to the tooth, and after its specific functions are performed it is doubtful whether the loss of the nerve is of any great moment. At least we all know of very many teeth from which the nerve has been removed and the canals properly filled that have been useful and without pain or discomfort for a score of years or more. Of course it is much to be prefered that there should be no exposed nerve, but this is not always within our control and we must be prepared to meet all cases as they are presented.

Though with children the inducement to save the pulp is stronger than with adults yet I think that in most cases the tooth can be saved to a longer period of usefulness by immediately destroying the exposed pulp than by striving to save it alive. In these days of ice-water and hot tea and biscuits the thermal changes to which the tooth is subjected are so great that even after treatment and careful capping there is a liability to pain and subsequent death of the nerves that under other circumstances might have lived. From this cause the terrible abscess and perhaps necrosis are often present before the patient is again met.

My conclusions are that it is always safer to destroy a fully exposed nerve, *cleanse* and *dry* the cavity and thoroughly fill. I hope to hear without reserve from those holding different opinions, and for this purpose alone has this hastily written paper been presented. It is possible that some who have investigated and perhaps met with different results in saving nerves alive, have some suggestions to make that will help us, at least in the cases of children and very young persons whose teeth would seem to be better with the saved nerve if the operation can be made an assured success.

